# Supplementary material for: Amino-Acid-Substituted Perylene Diimide as the Organic Cathode Materials for Lithium-Ion Batteries
Source: Materials (Basel). 2023 Jan 15;16(2):839. doi: 10.3390/ma16020839 (PMC9861502; doi:10.3390/ma16020839)
Supplement: Supplementary file 1 [file materials-16-00839-s001.zip › materials-2152613-supplementary.pdf]

Supplementary Material

Amino-Acid-Substituted Perylene Diimide as the Organic Cathode Materials for Lithium-Ion Batteries

Honggyu Seong †, Wonbin Nam †, Geongil Kim, Joon Ha Moon, Youngho Jin, Seung-Ryong Kwon, Joon-Hwa Lee * and Jaewon Choi *

Department of Chemistry and Research Institute of Natural Science, Gyeongsang National University, Jinju 52828, Republic of Korea

* Correspondence: joonhwa@gnu.ac.kr (J.-H.L.); cjw0910@gnu.ac.kr (J.C.).

† These authors contributed equally to this work.

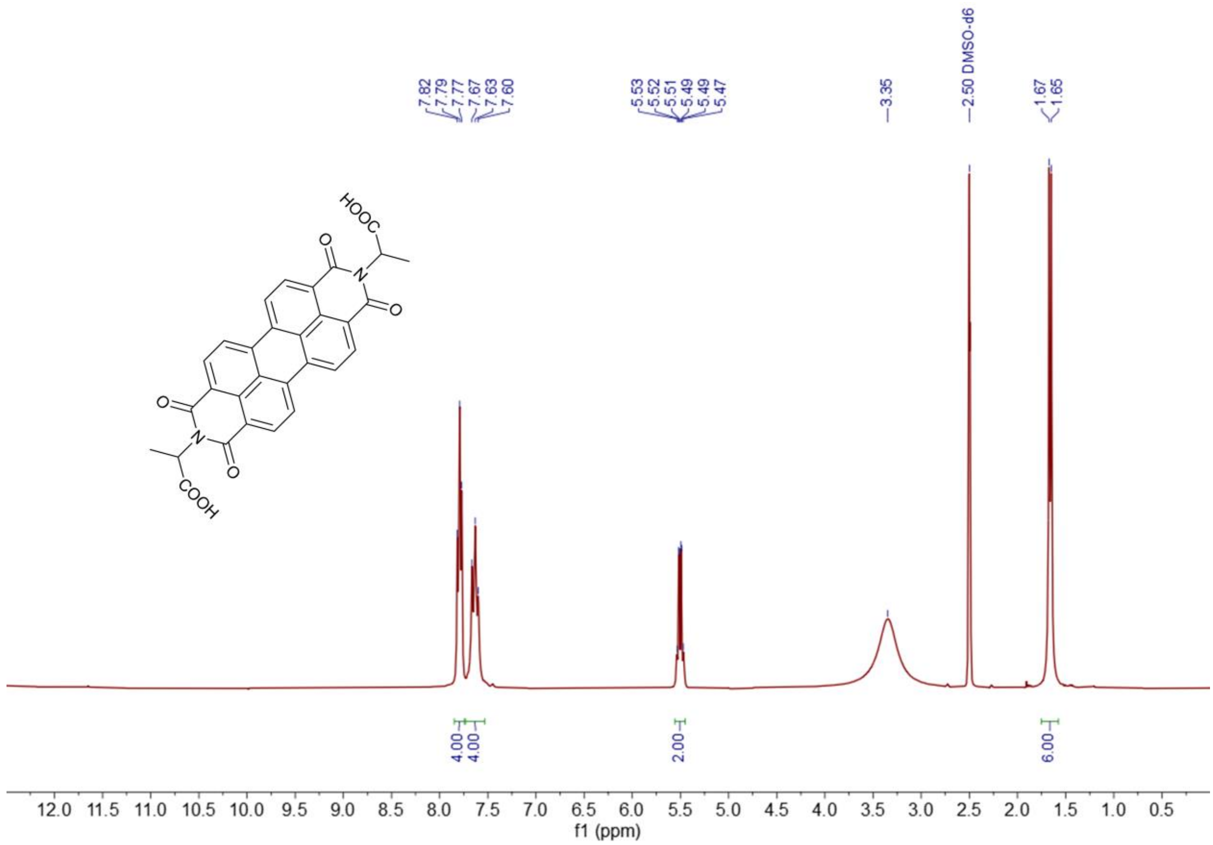

Figure S1. ^{1}H NMR spectrum (300 MHz, DMSO-d_6) of PDI_A

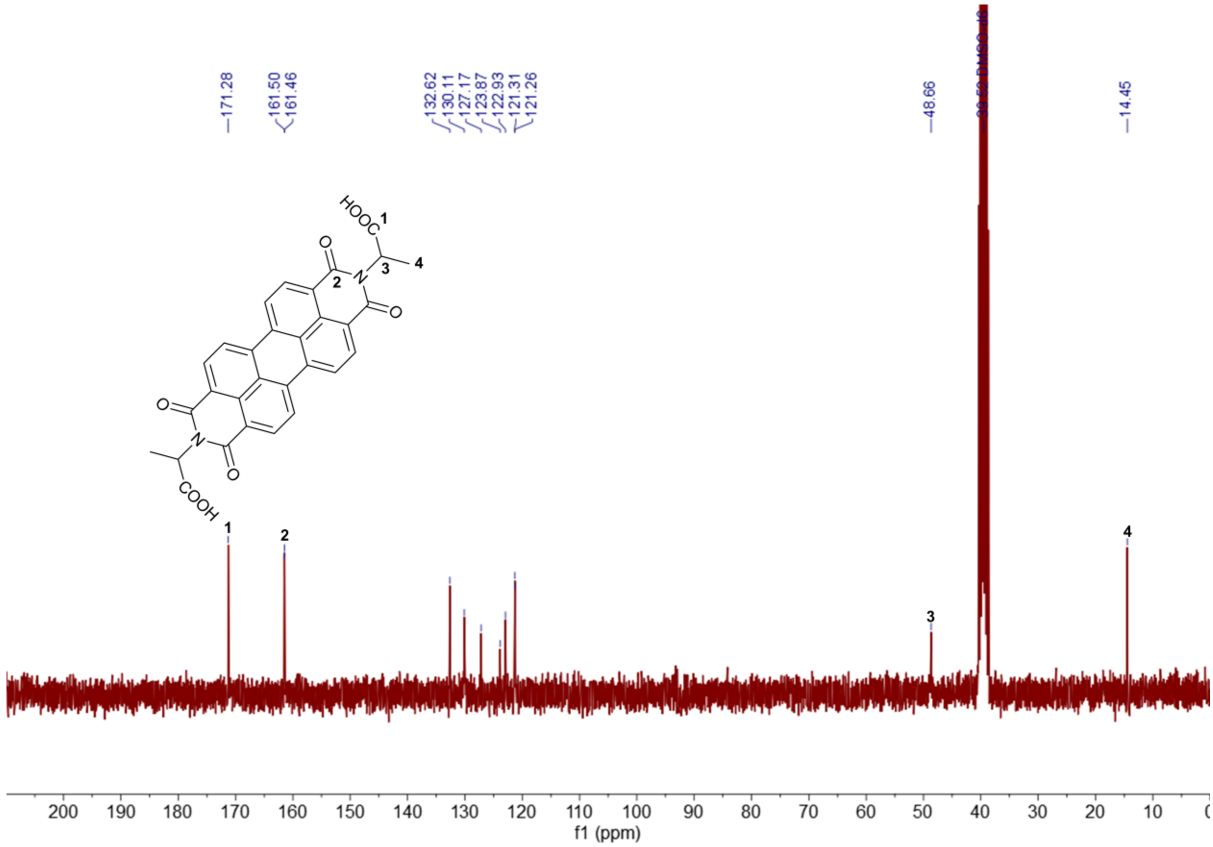

Figure S2. ^{13}C NMR spectrum (75 MHz, DMSO-d_6) of PDI_A

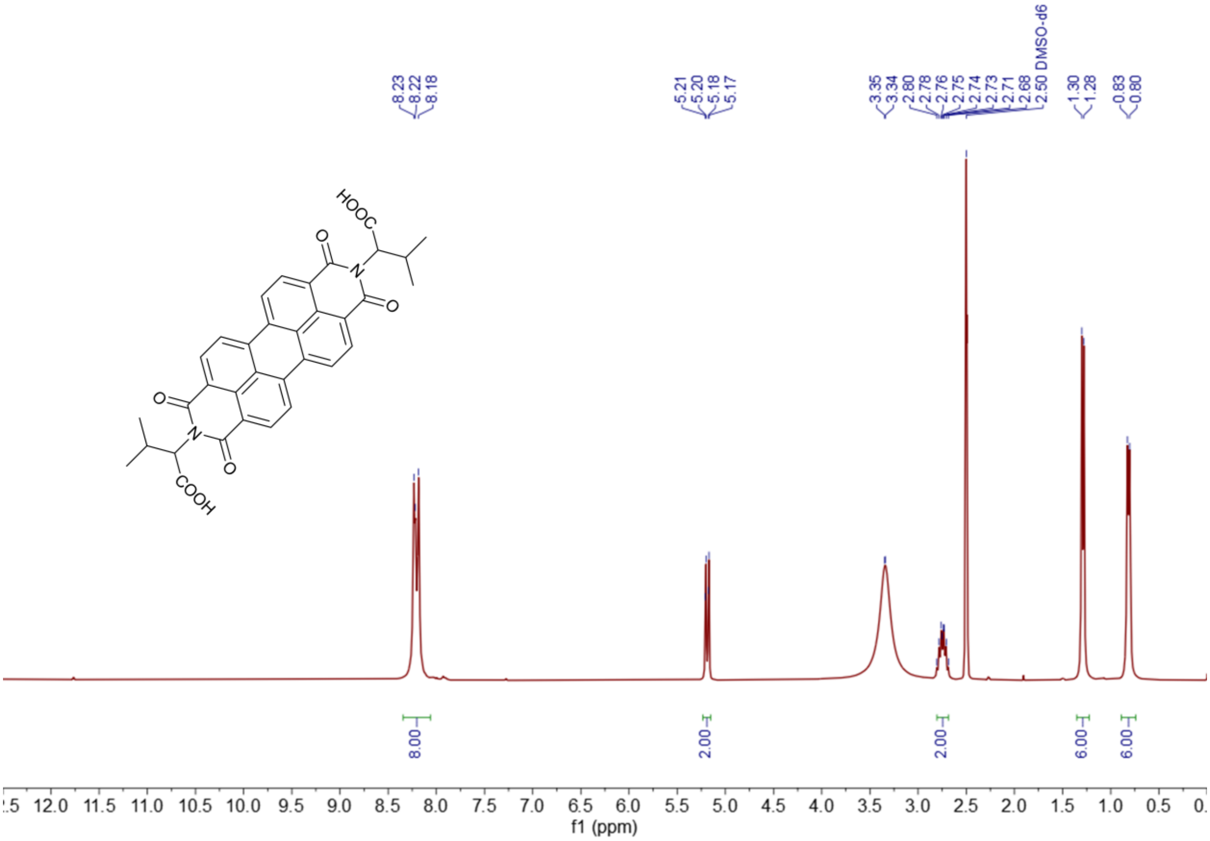

Figure S3. ^{1}H NMR spectrum (300 MHz, DMSO-d_6) of PDI_V

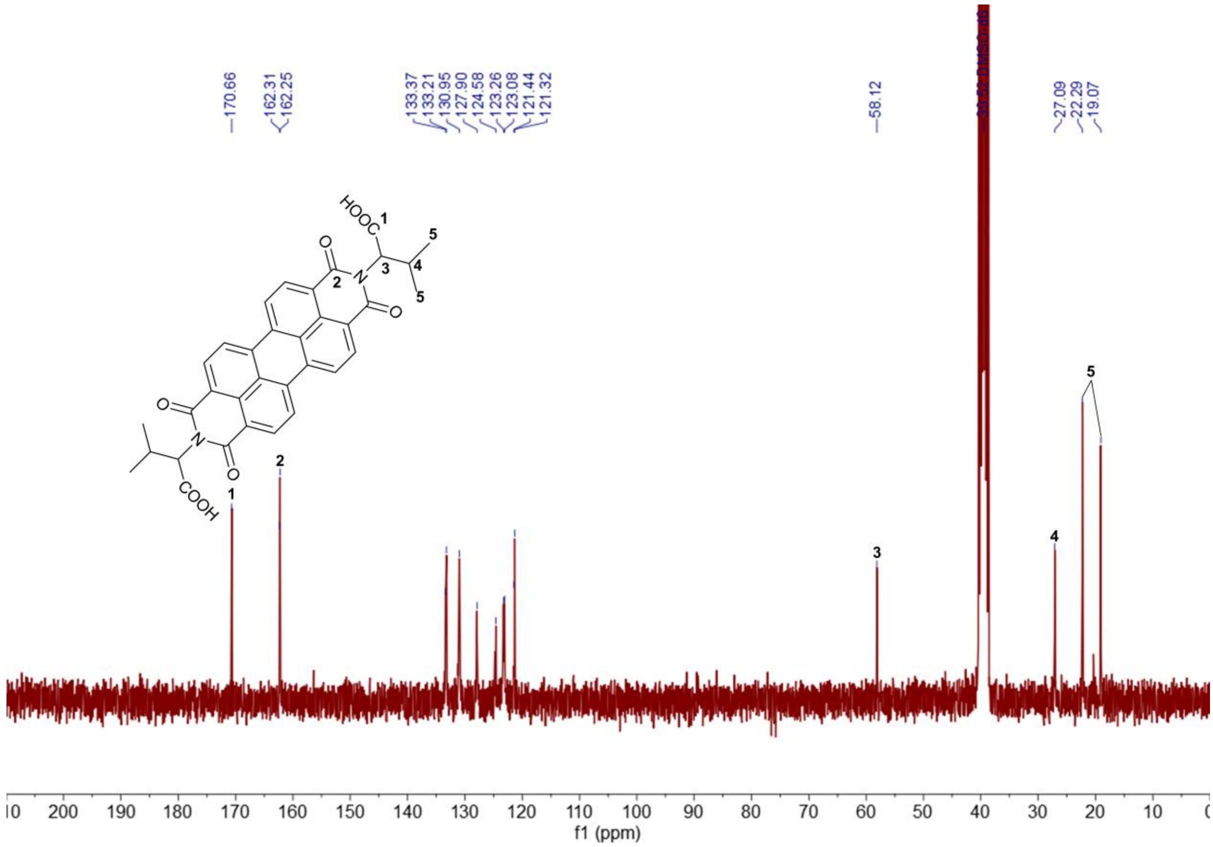

Figure S4. ^{13}C NMR spectrum (75 MHz, DMSO-d_6) of PDI_V

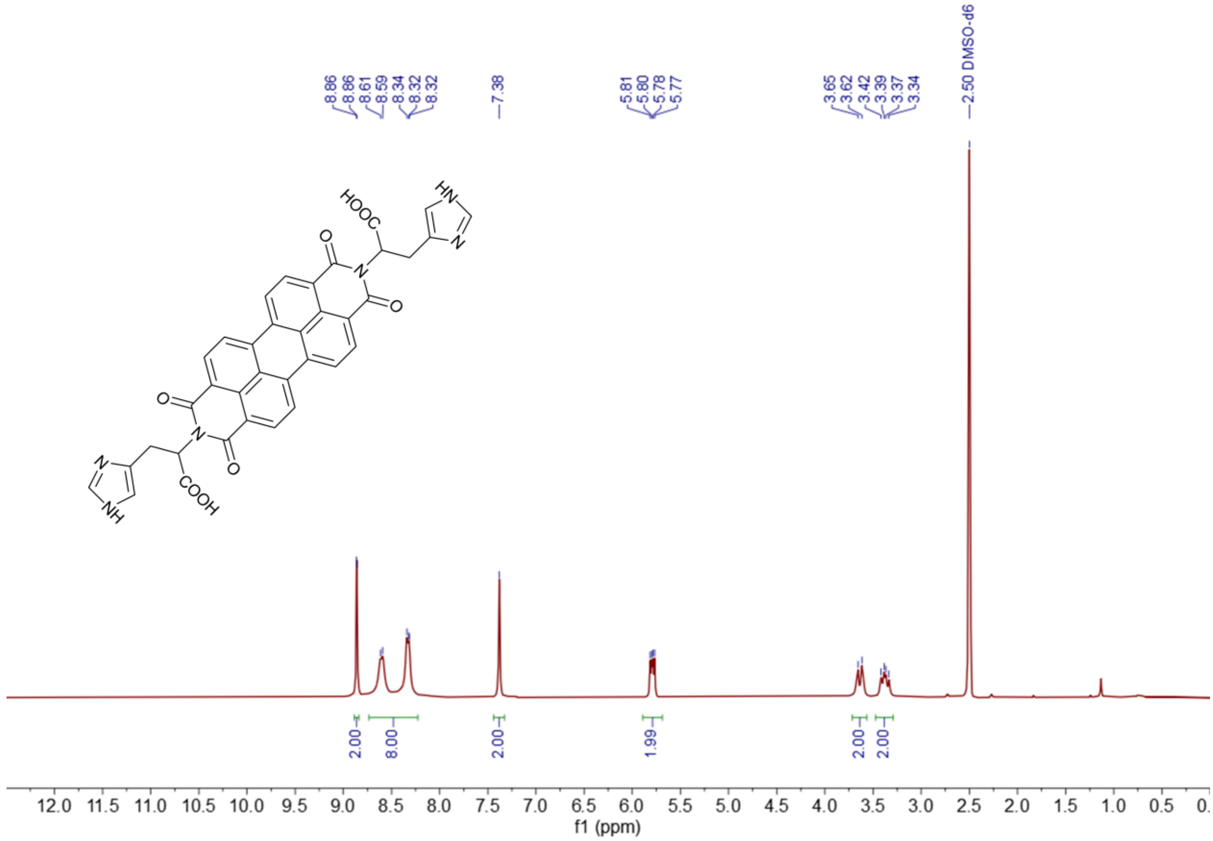

Figure S5. ^{1}H NMR spectrum (300 MHz, DMSO-d_6) of PDI_H

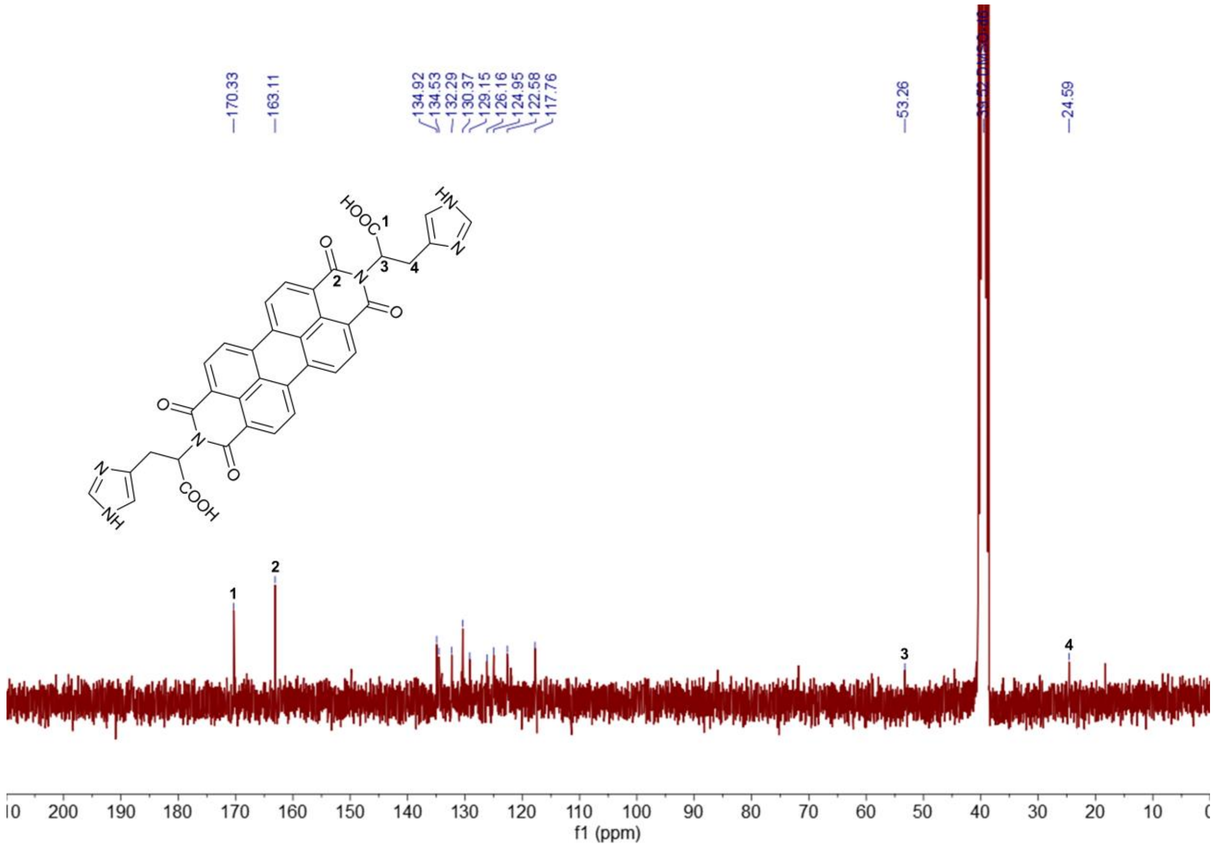

Figure S6. ^{13}C NMR spectrum (75 MHz, DMSO-d_6) of PDI_H

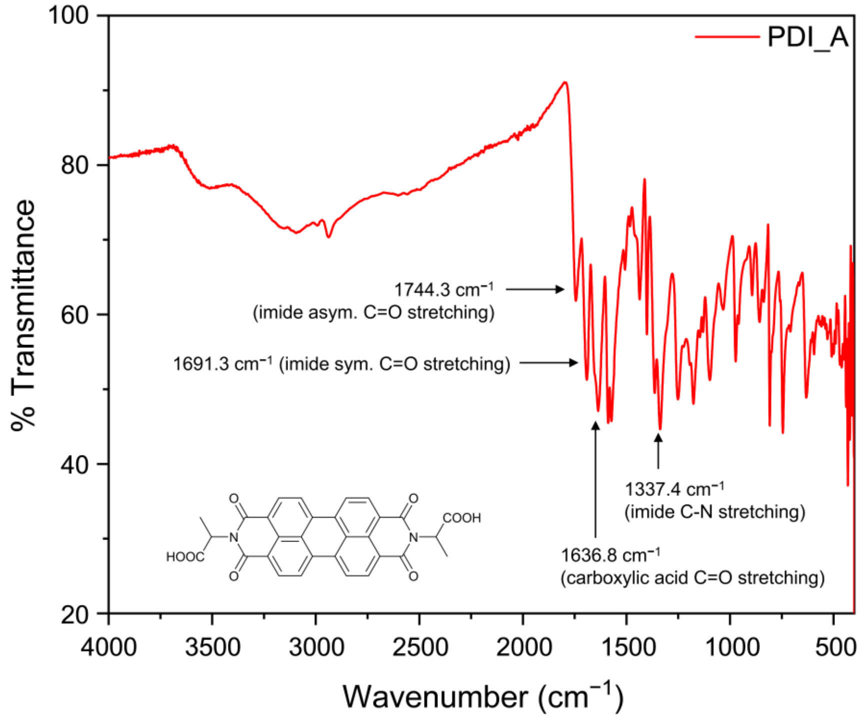

Figure S7. ATR FT-IR spectra of PDI_A

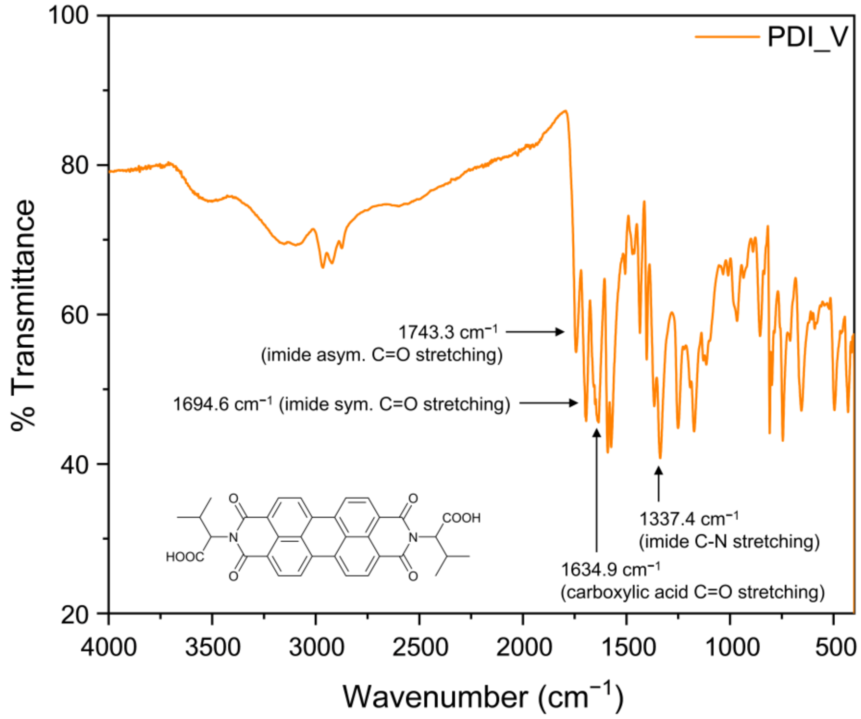

Figure S8. ATR FT-IR spectra of PDI_V

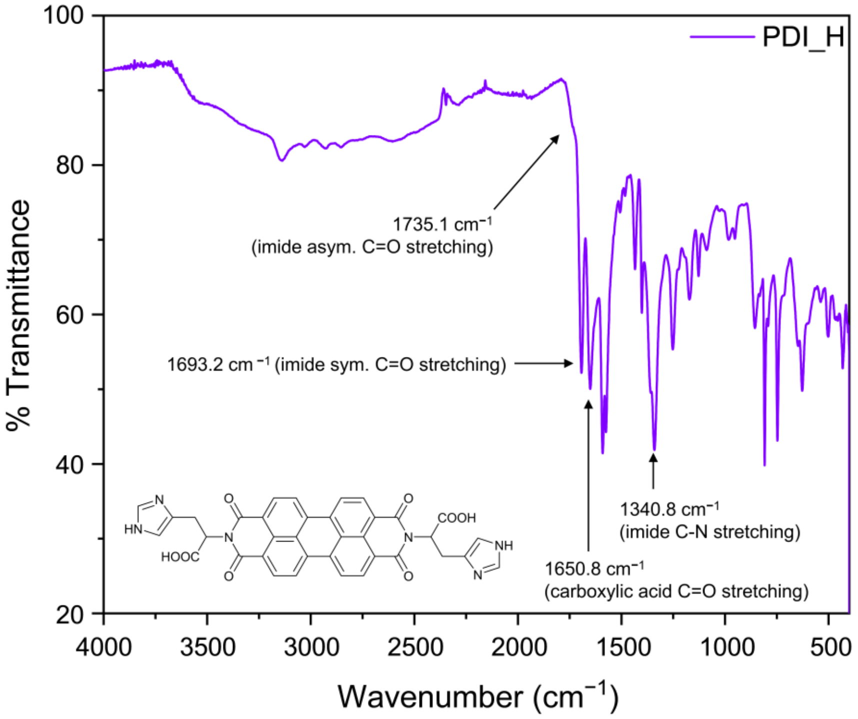

Figure S9. ATR FT-IR spectra of PDI_H

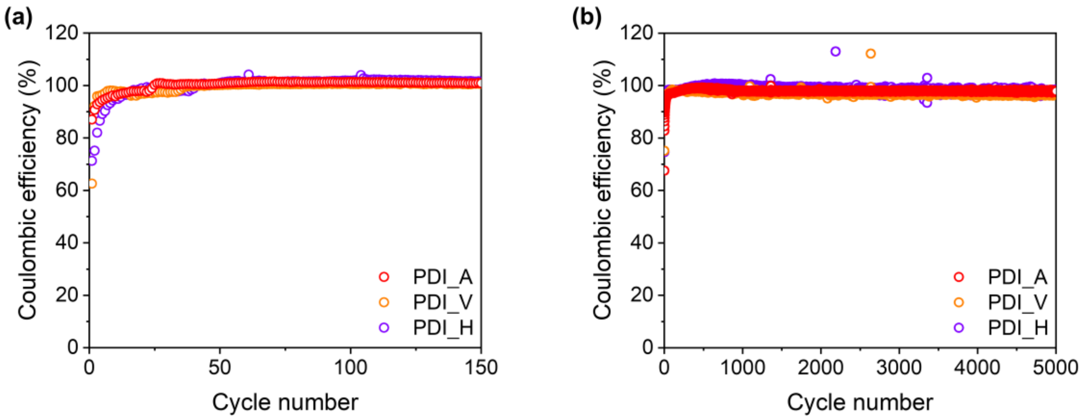

Figure S10. Coulombic efficiencies of PDI_AAs cathode at (**a**) 50 and (**b**) 500 mAg^{-1}

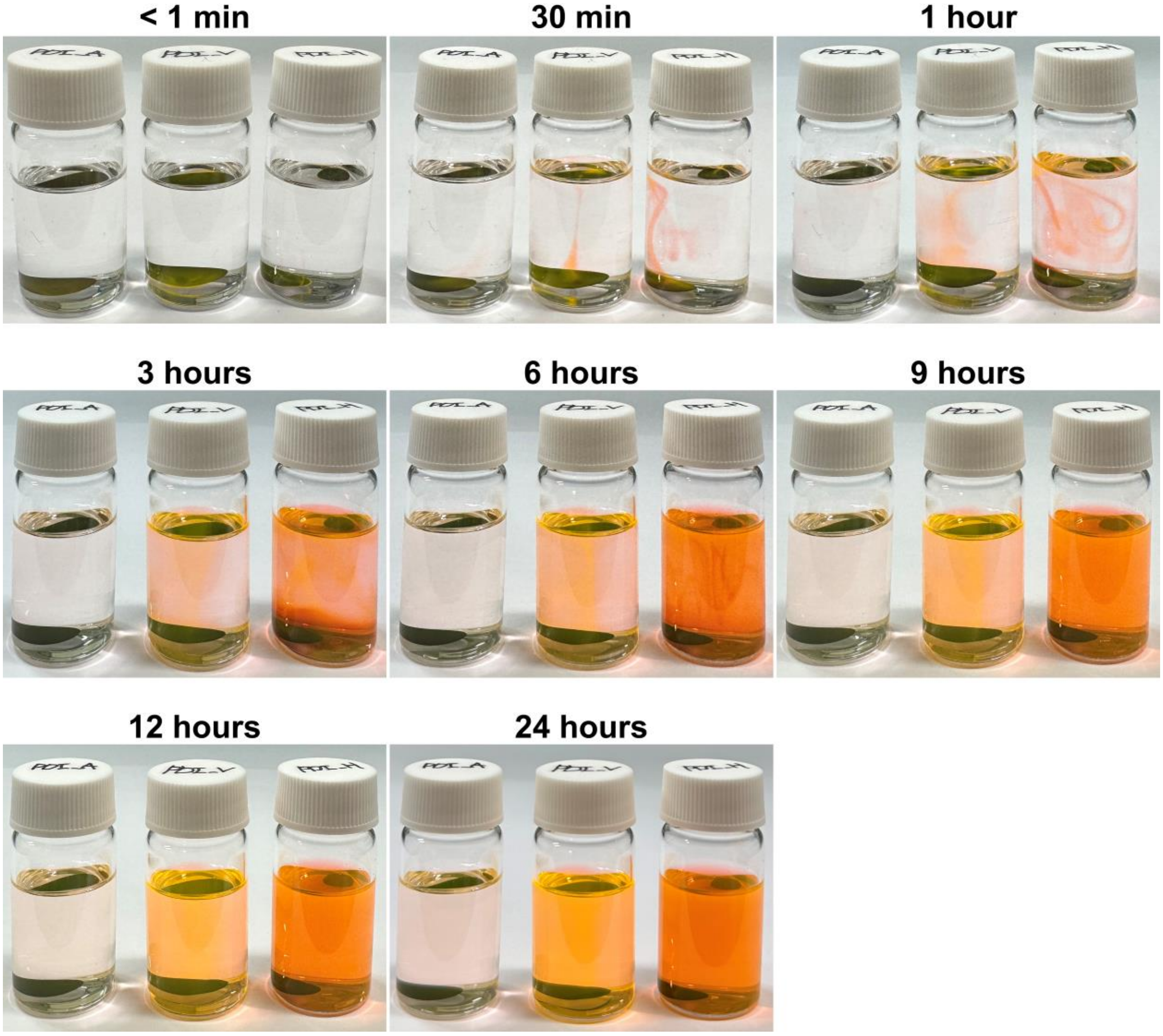

Figure S11. Photographs of PDI_AAs cathodes in electrolyte over time

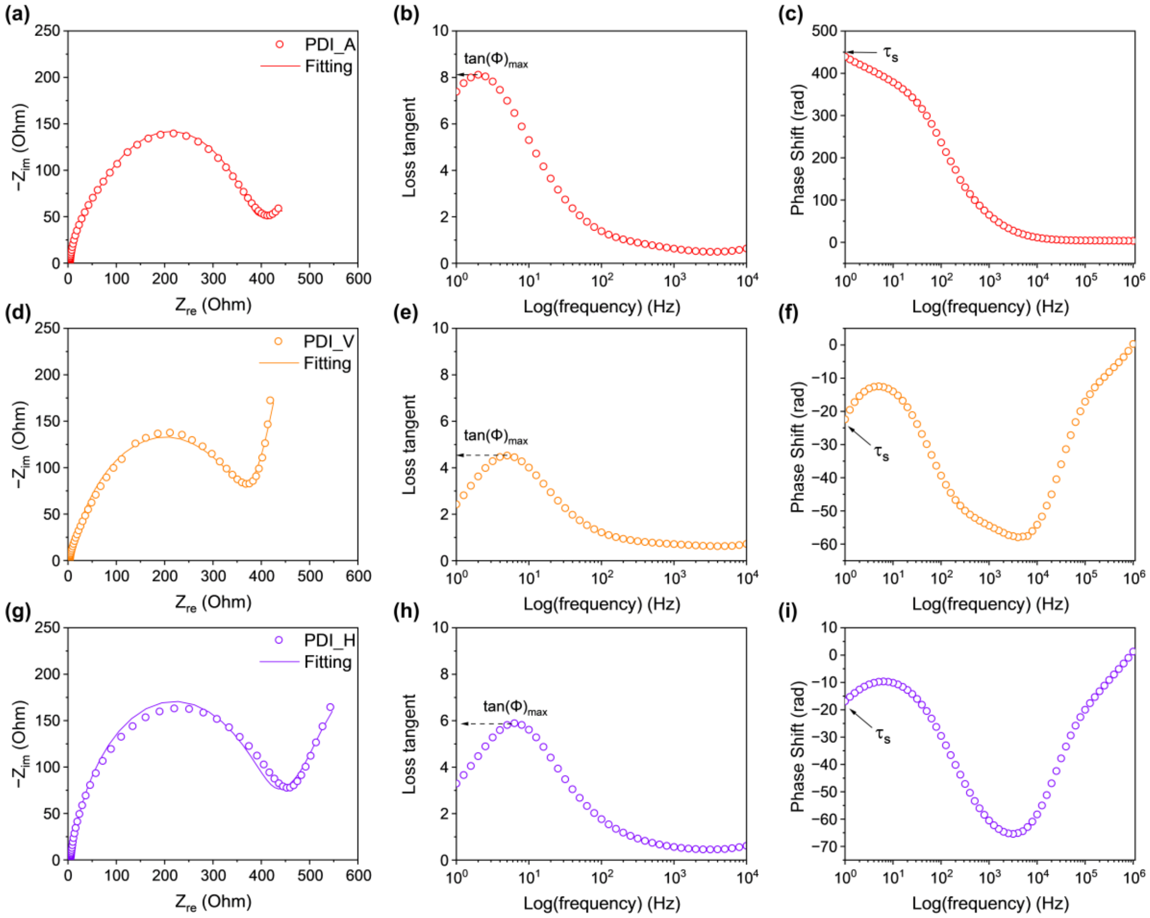

Figure S12. (**a**), (**d**) and (**g**) Nyquist plots. (**b**), (**e**) and (**h**) Bode plots. (**c**), (**f**) and (**i**) Loss tangent plots. (top: PDI_A; middle: PDI_V; bottom: PDI_H)

Table S1. Parameters for calculating Li-ion diffusion coefficient (D_{Li}) of PDI_AAs

Materials	τ_1 (s)	τ_s (s)	$\tan(\Phi)_{max}$	W_{sc} (s)	d (μm)	D_{Li} (cm^2s^{-1})
PDI_A	1.26×10^{-1}	1	8.12	3.96×10^{-1}	2.5	4.55×10^{-11}
PDI_V	1.58×10^{-1}	1	4.53	8.47×10^{-2}	2.5	8.18×10^{-9}
PDI_H	3.98×10^{-1}	1	5.89	9.90×10^{-1}	2.5	8.32×10^{-12}

We tried to set up a way to compare functional groups of PDI_A at different states. We needed a standard internal signal that should be unaffected by the solvation and maintain the bonding information during the charge/discharge process. Also, the as-selected internal standard signal should be distinct from the other absorption bands. Thus, we considered the perylene ring's two types of absorption bands as internal standard. %Transmittance obtained from ATR FT-IR (Table S2) is converted to absorbance (Table S3) through the relationship between them. We calculated the ratio of absorbance of the main functional group to the absorbance of perylene rings, respectively (Table S4, S5), which these ratios have the quantitative property. In Table S4 and S5, the ratios of C=O groups of PDI_A in the fully charged state are greater than in the discharged state. On the other hand, the ratios of the lithium enolate increase and slightly decrease again. The changes in these ratios are slightly small but indicate the formation of the lithium enolate coordinated carboxylic C=O and the reversible lithiation/delithiation process.

Table S2. %Transmittance of main functional group at different states of PDI_A

	**Imide C=O (asymmetrical stretch) (1748.2 cm^{-1})**	**Imide C=O (symmetrical stretch (1685.5 cm^{-1})**	**Carboxylic acid C=O (1637.3 cm^{-1})**	**Lithium enolate (1606.4 cm^{-1})**	**Perylene ring #1 (1585.7 cm^{-1})**	**Perylene ring #2 (1569.8 cm^{-1})**
pristine	44.9650	41.8281	38.6270	-	38.2324	37.9896
Discharged to 1.00 V	36.2239	30.3696	28.7588	28.1609	24.1762	23.2401
Charged to 4.00 V	19.7133	14.8286	12.9075	13.5636	10.4073	9.7625

Table S3. Absorbance of main functional group at different states of PDI_A

	**Imide C=O (asymmetrical stretch) (1748.2 cm^{-1})**	**Imide C=O (symmetrical stretch (1685.5 cm^{-1})**	**Carboxylic acid C=O (1637.3 cm^{-1})**	**Lithium enolate (1606.4 cm^{-1})**	**Perylene ring #1 (1585.7 cm^{-1})**	**Perylene ring #2 (1569.8 cm^{-1})**
Pristine	0.3471	0.3785	0.4131	-	0.4176	0.4203
Discharged to 1.00 V	0.4410	0.5176	0.5412	0.5504	0.6166	0.6338
Charged to 4.00 V	0.7052	0.8289	0.8892	0.8676	0.9827	1.0104

Table S4. Ratio(%) of the absorbance of main functional group to perylene ring #1 at different states of PDI_A

	**Imide C=O (asymmetrical stretch) (1748.2 cm^{-1})**	**Imide C=O (symmetrical stretch (1685.5 cm^{-1})**	**Carboxylic acid C=O (1637.3 cm^{-1})**	**Lithium enolate (1606.4 cm^{-1})**
Pristine	83.1302	90.6514	98.9319	-
Discharged to 1.00 V	71.5206	83.9362	87.7748	89.2544
Charged to 4.00 V	71.7683	84.3526	90.4847	88.2934

Table S5. Ratio(%) of the absorbance of main functional group to perylene ring #2 at different states of PDI_A

	**Imide C=O (asymmetrical stretch) (1748.2 cm^{-1})**	**Imide C=O (symmetrical stretch (1685.5 cm^{-1})**	**Carboxylic acid C=O (1637.3 cm^{-1})**	**Lithium enolate (1606.4 cm^{-1})**
Pristine	82.5832	90.0549	98.2809	-
Discharged to 1.00 V	69.5853	81.6650	85.3996	86.8392
Charged to 4.00 V	69.7954	82.0338	87.9972	85.8662
